# Immunohistochemical detection of laminin-1 and Ki-67 in radicular cysts and keratocystic odontogenic tumors

**DOI:** 10.1186/1472-6890-11-4

**Published:** 2011-03-02

**Authors:** Mohamed S Ayoub, Houry M Baghdadi, Moataz El-Kholy

**Affiliations:** 1 Professor, Oral Pathology Department, Faculty of Dentistry, Ain Shams University, Cairo, Egypt; 2 Associate Professor, Oral Pathology Department, Faculty of Dentistry, Ain Shams University, Cairo, Egypt; 3 Assistant Lecturer, Oral Pathology Department, Faculty of Dental Surgery, Modern Science and Arts University, Cairo, Egypt

## Abstract

**Background:**

Odontogenic cysts are those which arise from the epithelium associated with the development of teeth. Some odontogenic cysts were found to have special biological features that make them distinct from other lesions. This study was conducted to detect the immunoepxression of laminin-1 and Ki-67 in both radicular cysts (RCs) and keratocystic odontogenic tumors (KCOTs) and to examine the possible predictive value of these markers.

**Methods:**

Thirteen cases of RCs and twelve cases of KCOTs were included in this study. Antibodies against laminin-1 and Ki-67 were used as primary antibodies.

**Results:**

ten cases out of thirteen cases of RCs were immunopositive to laminin-1. The immunonegative cases of RCs showed high degree of inflammation inside the connective tissue wall. One case out of twelve cases of KCOTs was immunopositive to laminin-1 and the rest were immunonegative. Seven cases out of thirteen cases of RCs showed immunopositivity for Ki-67 with increased numbers of immunopositive cells when the inflammation was severe in the connective tissue wall. All KCOTS were immunopositive to Ki-67.

**Conclusions:**

The benign nature of radicular cysts and the aggressive behavior of keratocystic odontogenic tumors could be explained by the expression of laminin and Ki-67. Laminin-1 and Ki-67 could be valuable markers for the prediction of the biologic behavior of cystic lesions.

## Background

Radicular cysts are a direct sequel to chronic apical periodontitis following the death of dental pulp [1]. The epithelial rests of Malassez in periapical granuloma may be stimulated to proliferate by inflammatory stimuli [2]. The morphological aspects of the epithelium have been considered to reflect the functional activity of the RCs [3]. RCs depict a thin, regular and atrophic layer of stratified squamous epithelium, usually with mild to moderate inflammatory reaction [4]. The underlying supportive connective tissue might be focally or diffusely infiltrated with mixed inflammatory cells population [5].

Keratocystic odontogenic tumor (KCOT), previously known as odontogenic keratocyst (OKC), is a relatively common developmental odontogenic cyst that arises from the dental lamina remnants [6]. An important aspect of the OKC that should be underlined is that it can represent one component of the nevoid basal cell carcinoma syndrome (NBCS) [7]. Several studies have shown that the OKC is well recognized by its invasive potential [8], thus it tends to grow within the medullary cavity of bone and becomes a large lesion without causing obvious expansion [9].

Expression of laminin-1 in normal oral mucosa, odontogenic cysts and odontogenic tumors was examined in several studies. Sections of normal oral mucosa and odontogenic cysts stained for laminin-1 showed a distinct linear deposit of strong intensity at the basement membrane junction but not in the cytoplasm of the epithelial cells [10]. Sections of odontogenic tumors stained for laminin-1 showed strong reactivity at the basement membrane junction as well as in the cytoplasm of all tumor cells. The expression of laminin-1 in the cytoplasm of the tumor cells, but not in the normal mucosa may be a useful marker to distinguish these two types of epithelium [11] and it may suggest that laminin-1 influences the proliferation activity toward tumor potential [12].

Ki-67 antigen is the prototypic cell cycle related nuclear protein, expressed by proliferating cells in all phases of the active cell cycle (G1, S, G2 and M phase) and reaches a peak in the G2 and M phases. It rapidly degrades after mitosis with a half life of detectable antigen being an hour or less. It is absent in resting (G0) cells. Ki-67 antibodies are useful in establishing the cell growing fraction in neoplasms [13].

The aims of this study were to detect immunohistochemically the expression of laminin-1 and Ki-67 in radicular cysts and keratocystic odontogenic tumors and also to examine the possible predictive value of these markers.

## Method

### Specimen selection

Twenty-five formalin-fixed, paraffin-embedded tissue blocks of odontogenic cysts were obtained from the archives of the oral pathology departments, Ain Shams University, Alexandria University, and National Cancer Institute, Cairo University. Thirteen cases were diagnosed as radicular cysts (RCs) and twelve cases were diagnosed as keratocystic odontogenic tumors (KCOTs). Haematoxylin and eosin stained sections were used to confirm the diagnosis.

### Immunohistochemical procedures

For all specimens 4 μm sections were cut and mounted on positively charged glass slides. Sections were deparaffinized with xylene and rehydrated in graded ethyl alcohol, sections were immersed in citrate buffer solution of pH 4.8 and were put in the microwave oven before staining procedures.

For immunostaining a universal kit (R&D Systems; USA) was used, peroxidase anti- peroxidase method of immunostaining using the streptavidin-biotin system was carried out, 3% hydrogen peroxide was applied to the sections to block the endogenous peroxidase activity. The sections were immunostained with anti-laminin1 primary antibody (clone AL-2, R&D Systems, USA) and anti-Ki-67 primary antibody (clone BGX, Biogenix Corp., USA). The tissue sections were incubated overnight at room temperature. Sections were then covered by the link antibody followed by the streptavidin labeling antibody. After rinsing with PBS, DAB chromogen was applied to the sections followed by counter stain, and then sections were dehydrated in graded alcohol, cleared in xylene and mounted.

### Image analysis

For each positive section, four microscopic fields showing immunopositivity were selected and photomicrographs were captured at a magnification of 20×. Images were then transferred to the computer system for analysis using the image analysis software (Image J, 1.43r, NIH, USA), to determine the following:

1. Area fraction of immunopositivity for both laminin-1 and Ki-67. Area fraction was calculated as the ratio of immunopositive area to the total area of microscopic field.

2. Number of immunopositive cells for Ki-67

### Statistical Analysis

Statistical analysis was carried out on the tabulated data using (SPSS 16.0) software. The performed statistical tests included Student's T-test to compare between the expression of each marker in the two lesions and Pearson's correlation to determine the correlation between laminin-1 and Ki-67.

## Results

### I. Immunohistochemical Results

#### A) laminin-1

Lesions were considered positive when minimal brown staining was detected in the field. The immunopositivity at the basement membrane area of the blood vessels and in relation to inflammatory cells was excluded during analysis. The immunopositive reaction appeared as brown linear staining at the basement membrane of the epithelial cells.

**Figure 1:**
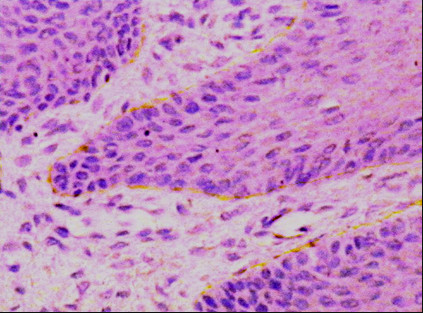
**Photomicrograph of RC showing discontinuous linear deposition of laminin-1 at the basement membrane area (laminin-1 ×200)**.

Ten cases out of the thirteen RCs were immunopositive to laminin-1 representing 76.9%. They showed a discontinuous linear deposition at the basement membrane of the lining epithelium (Figure 1). The three immunonegative cases showed variable degrees of inflammatory reaction in the connective tissue wall.

**Figure 2:**
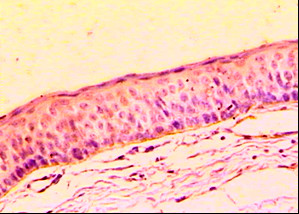
**Photomicrograph of KCOT showing continuous linear deposition of laminin-1 at the basement membrane area. (laminin-1 ×200)**.

One case out of the twelve cases of KCOTs revealed immunopositive reaction to laminin-1 representing 8.3%. The reaction appeared as a continuous linear deposition at the basement membrane area (Figure 2). Immunonegative reaction was observed in the remaining eleven cases of the KCOTs.

#### B) Ki-67

**Figure 3:**
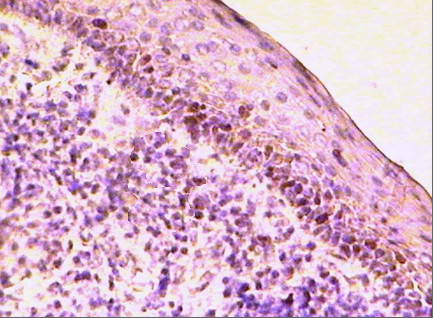
**Photomicrograph of RC showing Ki-67 immunopositive basal cells. Note the severe inflammatory reaction in the connective tissue (Ki-67 ×200)**.

Immunopositivity for Ki-67 appeared as brown reticular reaction confined to the nucleus. Seven cases of the thirteen RCs were immunopositive to Ki-67 representing about 53.8% where there was severe inflammation in the connective tissue. They showed a nuclear staining mainly in the basal cells. The number of immunopositive cells increased with the increased inflammatory reaction in the connective tissue (Figure 3). Immunonegative reactions were observed in the remaining six cases of the RCs.

**Figure 4:**
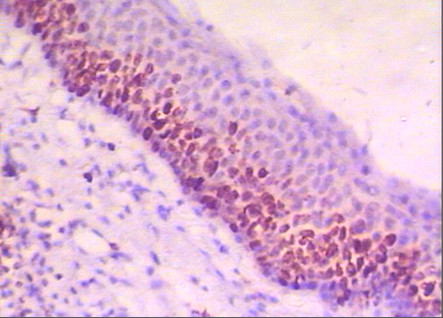
**Photomicrograph of KCOT showing Ki-67 immunopositvity mostly confined to the basal and suprabasal cells (Ki-67 ×200)**.

All cases of KCOTs were positive for Ki-67. The reaction was nuclear and confined to the basal and suprabasal cells of the epithelial lining (Figure 4).

### II. Statistical Results

Statistical analysis for lamini-1 immunoexpression in both RCs and KCOTs was not valid as laminin-1 was expressed in only one case of KCOT.

For Ki-67, keratocystic odontogenic tumor showed a statistically significant higher mean area fraction and higher mean number of Ki-67 immunopositive cells when compared with RCs (Tables 1, 2).

**Table 1:** The means, standard deviation (SD) values and results of Student's t-test for the comparison between Ki-67 area fraction in RC and KCOT

	**RC**		**KCOT**		
		
**Ki-67**	**Mean**	**SD**	**Mean**	**SD**	***P*-value**
	
	0.72	0.29	2.68	0.55	<0.001*

*: Significant at P ≤ 0.05

**Table 2:** The means, standard deviation (SD) values and results of Student's t-test for the comparison between number of Ki-67 +ve cells in RC and KCOT

	**RC**		**KCOT**		
		
**Number of +ve cells**	**Mean**	**SD**	**Mean**	**SD**	***P*-value**
	
	152	65.8	682.8	257.5	<0.001*

*: Significant at P ≤ 0.05

**Figure 5:**
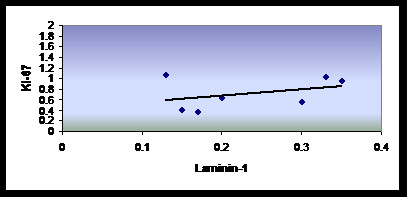
**Scatter plot showing positive correlation between Laminin-1 and Ki-67**.

On the other hand, statistical analysis with Pearson's correlation coefficient revealed a positive linear correlation between laminin-1 and Ki-67 immunopositive area fraction. However, in RCs, this correlation was not proven to be statistically significant. (Table 3 and Figure 5).

**Table 3:** Results of Pearson's correlation coefficient for the correlation between Lamini-1 and Ki-67 area fractions in radicular cysts

Correlation coefficient (r)	*P*-value
0.381	**0.399**

## Discussion

The results of the present study revealed that ten cases out of thirteen of RCs (76.9%) showed immunopositive expression of laminin-1 at the basement membrane zone of the epithelial lining. This is in accordance with the results of Poomsawat et al. [14]. Interestingly, the negative immunostaining for laminin-1 was observed in three RCs (23.1%) that showed severe inflammatory reaction in the connective tissue wall of the cysts. This result is in accordance to that reported by Furuyama et al., [15] who stated that the ability of epithelial cells to form continuous basement membrane was lost in the presence of inflammatory cytokines which enhances the secretion of matrix metalloproteinase (MMP-9) and (MMP-2).

On the other hand, negative immunoexpression of laminin-1 was observed in eleven cases out of twelve cases of OKCs included in this study. This finding is in agreement with the results of Amorim et al., [16]. In contrast Poomsawat et al., [14] concluded that laminin-1 was expressed in RCs, dentigerous cysts and odontogenic keratocyst with different distribution patterns and intensity. Also, Gurgel et al., [17] investigated the expression of laminin-1 in twenty cases of odontogenic keratocysts and found that laminin-1 was expressed in all cases.

Seven cases of the RCs included in the present study were immunopositive for Ki-67, which represent 53.8% of the total cases. The expression was confined mainly to the basal cells. Interestingly the surface area and the number of immunopositive cells increased with the severity of inflammation in the connective tissue. This could be explained on the assumption that chronic inflammatory reaction could act as stimulators causing epithelial proliferation. An explanation similar to that reported by Willoughby et al. [18] who concluded that mild inflammatory injury stimulates epithelial proliferation, whereas more severe inflammation depresses it, perhaps due to more extensive progenitor-cell damage.

Immunopositivity for Ki-67 was detected in all cases of KCOTs included in the study. The expression was mainly in the supra-basal cells, a finding similar to that reported by Kichi et al [19].

However, further studies utilizing a larger sample size and more advanced methodological tools are recommended due to limited number of cases included in this study.

## Conclusions

Based upon the results of the present study, it could be concluded that:

• The benign nature of radicular cysts and the aggressive behavior of keratocystic odontogenic tumors could be explained by the expression of laminin and Ki-67.

• Laminin-1 and Ki-67 could be valuable markers for the prediction of the biologic behavior of cystic lesions.

## Competing interests

The authors declare that they have no competing interests.

## Authors' contributions

MSA participated in the study design, photomicrography of the immunohistochemical results, interpreting and displaying the results of the study, carried out the sequence alignment and drafted the manuscript. **H.M.B **participated in displaying the results of the study, writing the discussion of the results and alignment of the references. ME carried out the immunohistochemical technique, collection of the background references and participated in writing the discussion of the results.

## Pre-publication history

The pre-publication history for this paper can be accessed here:

http://www.biomedcentral.com/1472-6890/11/4/prepub
